# Concerted functions of *Streptococcus gordonii* surface proteins PadA and Hsa mediate activation of human platelets and interactions with extracellular matrix

**DOI:** 10.1111/cmi.12667

**Published:** 2016-10-11

**Authors:** Jennifer A. Haworth, Howard F. Jenkinson, Helen J. Petersen, Catherine R. Back, Jane L. Brittan, Steve W. Kerrigan, Angela H. Nobbs

**Affiliations:** ^1^ School of Oral and Dental Sciences University of Bristol Bristol UK; ^2^ Cardiovascular Infection Group Royal College of Surgeons in Ireland Dublin 2 Ireland

## Abstract

A range of *Streptococcus* bacteria are able to interact with blood platelets to form a thrombus (clot). *Streptococcus gordonii* is ubiquitous within the human oral cavity and amongst the common pathogens isolated from subjects with infective endocarditis. Two cell surface proteins, Hsa and Platelet adherence protein A (PadA), in *S. gordonii* mediate adherence and activation of platelets. In this study, we demonstrate that PadA binds activated platelets and that an NGR (Asparagine‐Glycine‐Arginine) motif within a 657 amino acid residue N‐terminal fragment of PadA is responsible for this, together with two other integrin‐like recognition motifs RGT and AGD. PadA also acts in concert with Hsa to mediate binding of *S. gordonii* to cellular fibronectin and vitronectin, and to promote formation of biofilms. Evidence is presented that PadA and Hsa are each reliant on the other's active presentation on the bacterial cell surface, suggesting cooperativity in functions impacting both colonization and pathogenesis.

## 1 INTRODUCTION

*Streptococcus, Staphylococcus*, and *Enterococcus* bacteria account for >80% cases of infective endocarditis (Muñoz et al*.*, 2015; Slipczuk et al*.*, 2013) and are able to trigger activation or aggregation of blood platelets into a clot or thrombus (Fitzgerald, Foster, & Cox, 2006; Kerrigan, 2015). Viridans‐group streptococci that enter the bloodstream in otherwise healthy subjects almost always originate from the complex microbial communities present within the human oral cavity (Cahill & Prendergast, 2015; McNicol & Israels, 2010; Nilson, Olaison, & Rasmussen, 2015). Accordingly, there is a predictive link between levels of oral hygiene and the risk of cardiovascular disease (Lockhart et al*.*, 2009). Platelet adhesion and activation by oral streptococci occurs by several different mechanisms (Cognasse et al*.*, 2015; Kerrigan & Cox, 2010; McNicol, 2015). For *Streptococcus gordonii*, *S. oralis* and *S. sanguinis*, a common primary interaction occurs with platelet integrin receptor GPIb mediated by a bacterial surface serine‐rich repeat protein (Deng et al*.*, 2014). In *S. sanguinis*, a direct interaction between serine‐rich repeat protein SrpA and GPIbα leads to platelet rolling over immobilized bacteria and adhesion at low shear (Kerrigan et al*.*, 2002; Plummer et al*.*, 2005). *S. gordonii* serine‐rich repeat glycoprotein designated Hsa (Takahashi, Konishi, Cisar, & Yoshikawa, 2002), or GspB (Bensing & Sullam, 2002), seems to act in a similar manner (Bensing, López, & Sullam, 2004; Kerrigan et al*.*, 2007). Hsa and GspB recognize structurally‐distinct sialic acid‐oligosaccharides on GPIb (Takamatsu et al*.*, 2005). Once engaged with the streptococci the platelets become activated, spread, aggregate, and initiate thrombus formation, under which conditions, the bacteria are well protected from host immune defences and antibiotics (Jenne & Kubes, 2015; Jung et al*.*, 2015).

**Table 1:** List of primers. Restriction enzyme sites are underlined

Primer	Sequence
aad9.seqF	5′ GGATACATTCCTCAGAAGGAA 3’
padA.F1	5′ GGCCTCGAGCGGAATTTAACTAGGAGGG 3’
padA.R1	5′ CCTGGACGGCGCCCATGCTATTTTAAAGCCTATATAA 3’
padA.F2	5′ TAGCATGGGCGCCGTCCAGGAGAGCTATGCTCTT 3’
padA.R2	5′ TCCCCCCGGGGTCGTAATCTCTGGTTGAGG 3’
aad9F.NarI	5′ CGCGGCGCCCGTAACGTGACTGGCAAG 3’
aad9R.NarI	5′ CGCGGCGCCGATGCATATGCATGCTGC 3’
Terminator.XhoF	5′ ATGATACATGCTCGAGGAATTAGGTTGAAAAAATAGAAGG3’
Terminator.XhoR	5′ GGAGACCGGCCTCGACTGAACACAAAGCTCGTAAGATC 3’
padA.pMSPF	5′ ATGATACATGCTCGAGGATTTTTTGAAAAAGGTTTTAATAC 3’
padA.pMSPR	5′ AACCTAATTCCTCGAGTTAATGCTTTGGTTTTCTTCTC 3’
JH5F	5′PHO‐ GCTGCTGCAATCACACAGACCGGTAACAAGG 3′
JH6R	5′PHO‐GATTTCCTTGCTAGGGTCTGTTACTTTCGG 3′
JH19F	5′PHO‐ GCGGCCGCTAAGACGGTCTTCCTCCTAGTAACGG 3’
JH20R	5′ PHO – TTCCATATTCCCTTTGACACGGTTGTACTGAGC 3’
His.padAR	5′ ATGATGATGTGCTCCGTCTTTAATAGATG
His.padAF	5′ CACCACCACTAACTCGAGGAATTAGGTTG

The mechanisms by which platelets are activated by streptococci following adhesion are poorly understood. Recent work has identified the importance of specific antibodies in bacterial activation of platelets through receptor FcγRIIa (Arman et al*.*, 2014; Pampolina & McNicol, 2005; Tilley et al*.*, 2013). In *S. gordonii*, a ubiquitous human oral bacterium, there is direct activation of FcγRIIa integrin (non‐antibody mediated), and then inside‐out activation of the most highly‐expressed platelet receptor GPIIbIIIa (α_IIb_β_3_) (Keane et al*.*, 2010). Further activation is associated with secretion of granules and secondary mediators, filopodia formation (spreading), generation of ADP and thromboxane A2 (TxA2) to reinforce α_IIb_β_3_ activation (Cox, Kerrigan, & Watson, 2011), and then aggregation with nearby‐activated platelets via fibrinogen (Moriarty et al*.*, 2015).

**Figure 1:**
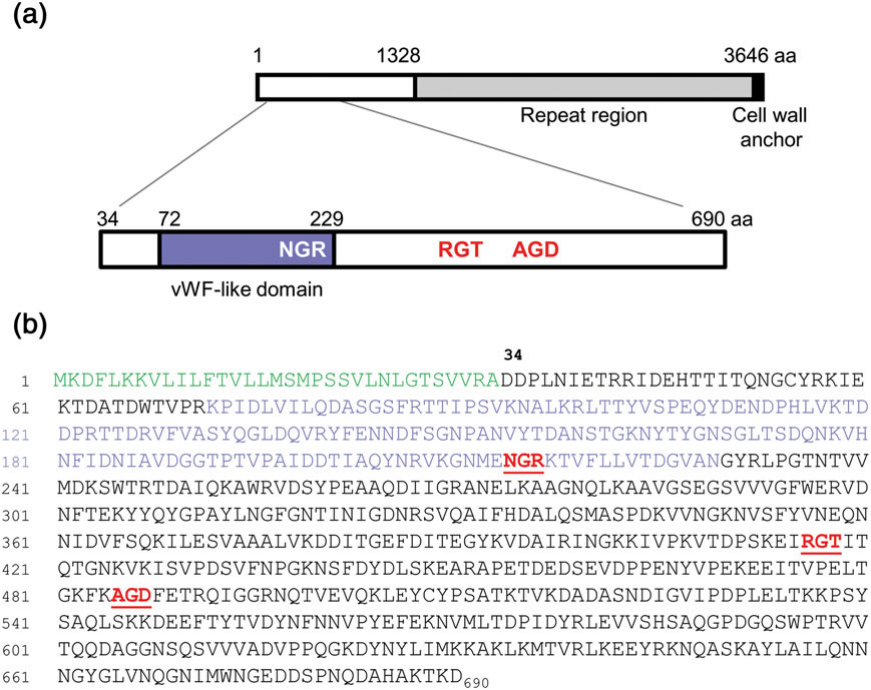
Diagrammatic representation of PadA protein and N‐terminal F2 domain. (a) PadA precursor comprises 3,646 aa residues with N‐terminal region (1,328 aa), and C‐terminal region (2,318 aa) carrying 14 repeat blocks of 148–152 aa residues and a cell wall anchor motif LPKTG. The F2 region of the PadA polypeptide (657 aa) has been expanded to show the extent of the vWF‐like domain and the approximate positions of NGR, RGT, and AGD motifs. (b) aa sequence of the N‐terminal region 690 aa residues, indicating signal (leader) sequence (in green), the vWF‐like domain (purple), and the three integrin‐recognition motifs, NGR (214–6 aa), RGT (416–8 aa), and AGD (485–7 aa) in red type

We have identified a protein designated Platelet adherence protein A (PadA), that is expressed on the surface of *S. gordonii*, and which interacts directly with platelets (Petersen et al*.*, 2010). PadA precursor (3,646 amino acid [aa] residues) comprises a N‐terminal region of 1,328 aa residues containing a von Willebrand Factor (vWF)‐like domain (aa residues 72–229), and a C‐terminal region comprising 14 blocks of aa residue repeats (Petersen et al*.*, 2010) with a bacterial cell wall anchor region and sortase (LPxTG) motif (Mazmanian, Ton‐That, & Schneewind, 2001) (see Figure 1). PadA, unlike Hsa, does not interact with sialylated GPIb (Petersen et al*.*, 2010), but was shown to bind directly to the platelet receptor α_IIb_β_3_ over‐expressed on the surface of Chinese hamster ovary cells (Petersen et al*.*, 2010). Binding of PadA to α_IIb_β_3_ on platelets results in platelet activation; however, it is not known if PadA is able to activate platelets on its own, or if it requires a coactivator. Previous results suggest that α_IIb_β_3_ may bind to *S. gordonii* via common integrin‐recognition motifs (RGT and AGD) present within the N‐terminal region of PadA (Keane et al*.*, 2013). Recently, it has emerged that another motif, NGR (Asn‐Gly‐Arg), found in the D domain of fibrinogen on each of the β and γ chains, plays an important role in the interaction of α_IIb_β_3_ with fibrinogen (Moriarty et al*.*, 2015). A similar motif is found within the N‐terminal region of PadA, raising the possibility that this might be a key factor in recognition of α_IIb_β_3_ by PadA.

In this article, we have investigated the relative functions of Hsa and PadA in *S. gordonii* platelet interactions, and in bacterial cell binding to various extracellular matrix components that may be exposed at sites of endothelial damage, for example, fibronectin or vitronectin. We have also studied in more detail the interactions of the mature N‐terminal F2 region (657 aa residues) of PadA with platelets and matrix components, and more specifically the relative roles of the integrin‐recognition motifs and NGR in *S. gordonii* host interactions.

## 2 RESULTS

### 2.1 Expression of *padA* and *hsa* in deletion mutants and complemented strains

**Figure 2:**
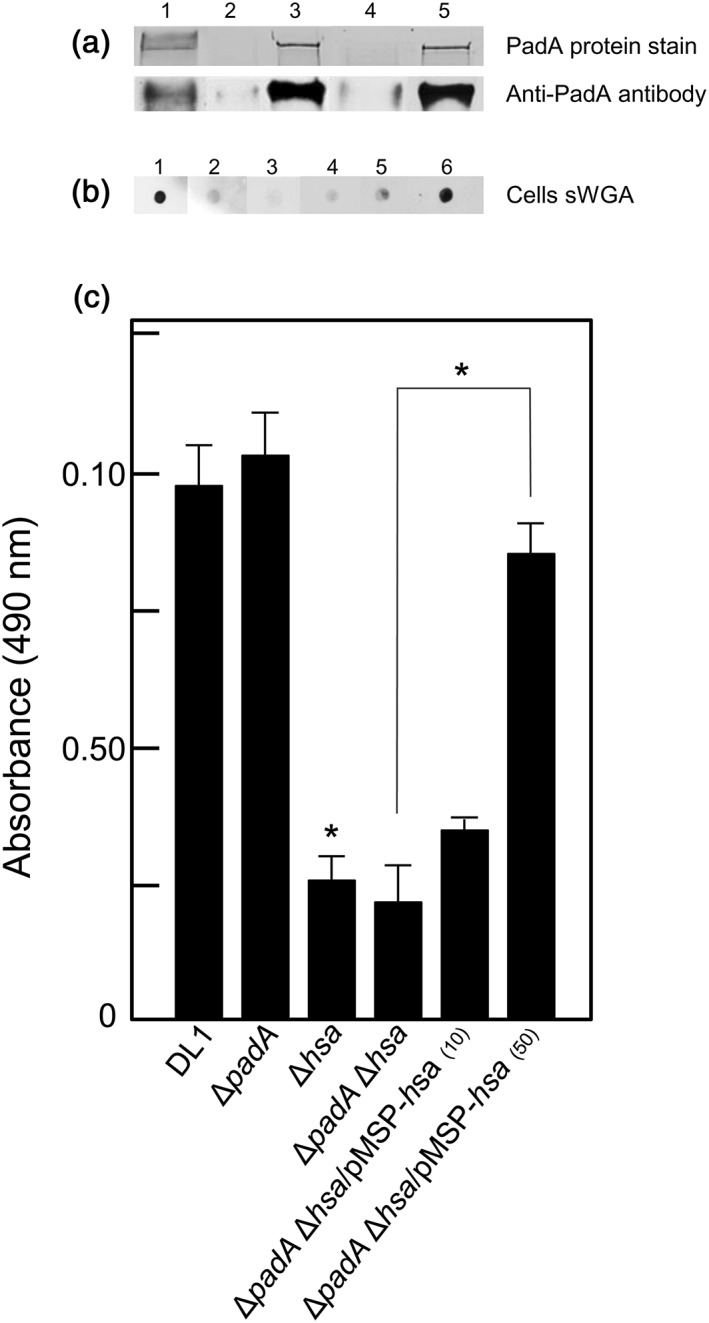
Expression of PadA and Hsa proteins by *S. gordonii* DL1 wild type, mutants and complemented mutants. (a) (composite), SDS‐PAGE gel, and corresponding Western immunoblot of PadA (~350 kDa) in cell wall proteins extracted from: (1) DL1; (2) UB2723 Δ*padA*; (3) UB2724 Δ*padA*/pMSP‐*padA*; (4) UB2773 Δ*padA* Δ*hsa*; and (5) UB2775 Δ*padA* Δ*hsa*/pMSP‐*padA.* In lanes 3 and 5, protein expression was induced with 10 ng nisin ml^−1^. (b) (composite), whole cell dot blots reacted with sWGA of: (1) DL1; (2) UB2029 Δ*hsa*; (3) UB2773 Δ*padA* Δ*hsa*; (4) UB2777 Δ*padA* Δ*hsa*/pMSP‐*hsa*; (5) UB2777 + 10 ng nisin ml^−1^; and (6) UB2777 + 50 ng nisin ml^−1^. (c) Biotinylated sWGA binding to immobilized bacterial cells (2 × 10^7^ per well) followed by HRP‐streptavidin to detect Hsa as described in 4. Figures in parentheses indicate ng ml^−1^ nisin added to cultures to induce expression of Hsa in the complemented strain UB2777 Δ*padA* Δ*hsa*/pMSP‐*hsa.* Error bars represent ±SEM from two independent experiments (*n* = 2). * *P* < 0.05

A diagrammatic representation of PadA protein is presented in Figure 1 together with a visual expansion of the 690 aa‐residue precursor N‐terminal domain (F2) of main focus in this paper, and the corresponding aa sequence. Three integrin‐like recognition motifs within the F2 domain are highlighted. To investigate further the functional properties of the PadA protein, the entire coding region was cloned into replicative plasmid vector pMSP downstream of a nisin‐inducible promoter (see 4). The *hsa* gene was cloned in the same way as previously described (Jakubovics, Brittan, Dutton, & Jenkinson, 2009). Because Hsa is glycosylated by various products encoded by genes within the *hsa*‐accessory secretion system locus (Zhou & Wu, 2009), authentic Hsa protein has not been expressed in a surrogate host bacterium. The results obtained from PadA Western immunoblot analyses of mutant and complemented strains are shown in Figure 2a. PadA protein was deficient in cell wall extracts of the Δ*padA* and Δ*padA* Δ*hsa* mutants, and was highly‐expressed in the complemented strains following 10 ng nisin ml^−1^ induction. Hsa production was detected with succinylated‐wheat germ agglutinin (sWGA), which recognizes the glycosylated protein (Takahashi, Yajima, Cisar, & Konishi, 2004). Hsa was well‐expressed ectopically in the complemented Δ*hsa* and Δ*padA* Δ*hsa* mutants when induced with 50 ng nisin ml^‐1^ (Figure 2b,c). Higher nisin concentrations were growth‐inhibitory. Production of Hsa from the chromosomal locus in the Δ*padA* mutant was unaffected and was similar to wild type strain DL1 expression levels (Figure 2c; Petersen et al*.*, 2010).

### 2.2 Role of PadA and Hsa in platelet adhesion by *S. gordonii*

**Figure 3:**
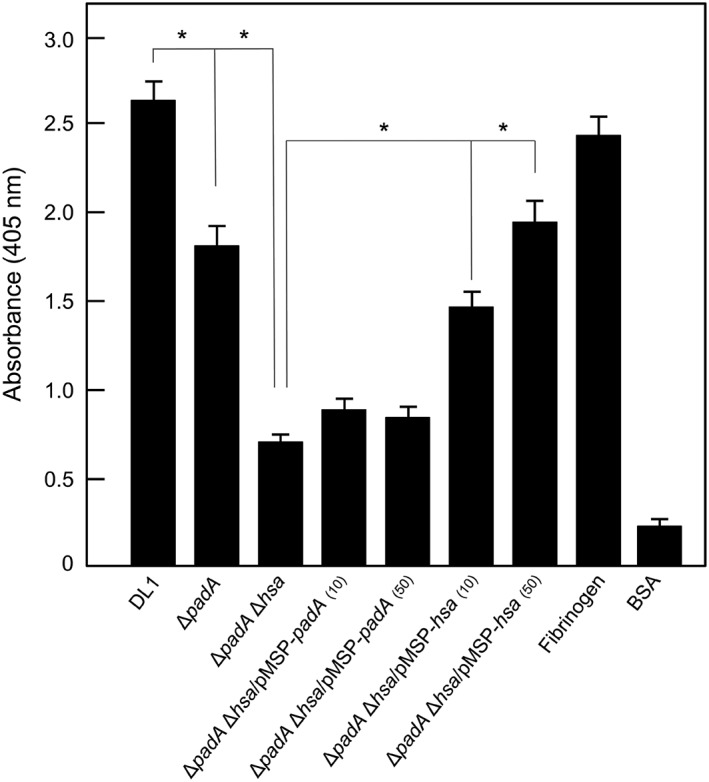
Platelet adhesion to immobilized *S. gordonii* strains under static conditions. Bacterial cells were deposited onto microwells, and platelet binding was determined by phosphatase assay as described in 4. Figures in parentheses indicate ng ml^−1^ nisin added to cultures to induce expression of PadA or Hsa in the complemented strains UB2775 Δ*padA* Δ*hsa*/pMSP‐*padA*, and UB2777 Δ*padA* Δ*hsa*/pMSP‐*hsa*. Fibrinogen 100 μg ml^−1^ positive control; BSA 100 μg ml^−1^ negative control. Error bars represent ±SEM from four independent experiments (*n* = 4). * *P* < 0.05

It is well documented that Hsa interacts with platelets (Keane et al*.*, 2010; Kerrigan et al*.*, 2007; Takahashi et al*.*, 2004), and we have previously shown that PadA also is involved in *S. gordonii* binding platelets (Petersen et al*.*, 2010). This is confirmed (Figure 3) with the Δ*padA* mutant being approximately 30% reduced in levels of platelet adherence, and the Δ*padA* Δ*hsa* mutant >80% reduced in binding platelets. Expression of *padA* in the Δ*padA* Δ*hsa* mutant does not restore any level of platelet adhesion, but expression of *hsa* restored platelet binding levels to ~70% of wild type (Figure 3). These results show that interaction of PadA with resting platelets requires the presence of Hsa.

### 2.3 PadA integrin‐recognition motifs are essential for binding platelets

**Figure 4:**
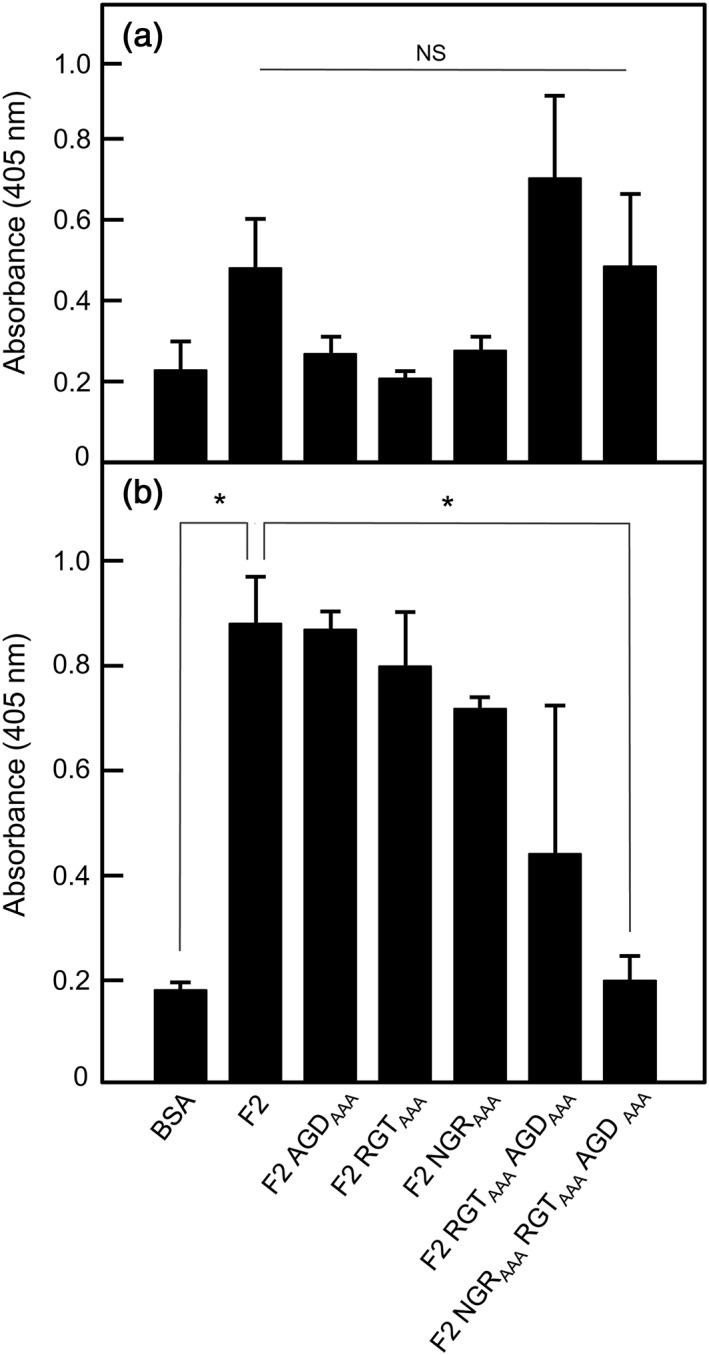
Adhesion of non‐activated or TRAP‐activated platelets to immobilized recombinant PadA‐F2 region fragments under static conditions. Recombinant PadA protein fragments were immobilized onto microtitre plate wells (10 μg per well), and non‐specific binding sites were blocked with BSA. Gel‐filtered platelets were either non‐activated (a) or activated by addition of TRAP (Thrombin Receptor Activating Peptide; b) and were incubated with the immobilized proteins at 37°C for 45 min (2 × 10^7^ platelets per well). Platelet adherence was determined by phosphatase assay. RGT_AAA_, AGD_AAA_, NGR_AAA_, and so forth indicate the motifs within the various F2 fragments that were alanine‐substituted to AAA. Error bars represent ±SEM from three independent experiments (*n* = 3). * *P* < 0.05. In (a), NS = not statistically significant (F2 v F2 RGT_AAA_ AGD_AAA_, *P* = 0.429; F2 v F2 NGR_AAA_ RGT_AAA_ AGD _AAA_, *P* = 0.962)

The above results suggest that Hsa and PadA might participate in a two‐step reaction with platelets. We envisage that Hsa interacts with GPIb (Bensing et al*.*, 2004) resulting in inside‐out signalling, activation of α_IIb_β_3_, and PadA interacting with activated α_IIb_β_3_ (Keane et al*.*, 2013). In support of this hypothesis, a *padA* mutant was unaffected in binding purified α_IIb_β_3_ but was abrogated in binding α_IIb_β_3_ on the surface of Chinese hamster ovary cells (Petersen et al*.*, 2010). The purified PadA‐F2 fragment (Figure 1) binds weakly to non‐activated platelets (Figure 4a) but at high levels to TRAP‐activated platelets (Figure 4b). We were interested in determining the role of the NGR motif within PadA‐F2 region because this same motif is present in fibrinogen and has been shown to interact with α_IIb_β_3_ and activate platelets (Moriarty et al*.*, 2015). Alanine substitutions of integrin‐like recognition motifs NGR, RGT, or AGD, to AAA in each case, individually had no significant effects on binding of F2 fragment to activated platelets (Figure 4b). However, when all three motifs were substituted, activated platelet binding was abrogated (Figure 4b) while non‐activated platelet binding was not significantly different from F2 (Figure 4a). Therefore, NGR is not only essential for interaction of PadA with activated platelets but also requires functional AGD and RGT motifs (Figure 4b).

**Figure 5:**
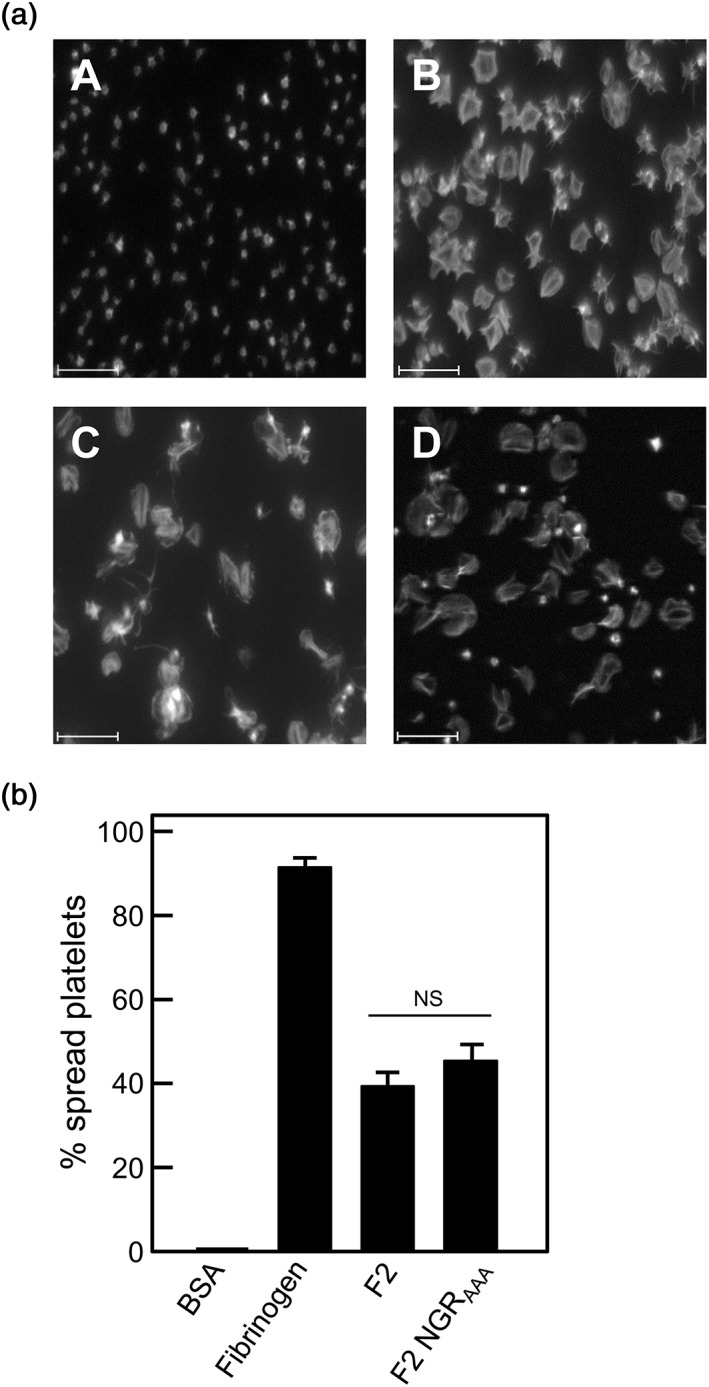
Platelet spreading on immobilized recombinant PadA‐F2 region fragments under static conditions. (a) BSA (negative control), (b) fibrinogen (positive control), (c) recombinant PadA protein fragment F2, or (d) fragment F2 NGR_AAA_ were immobilized onto microtitre plate wells (10 μg per well), and non‐specific binding sites were blocked with BSA. Gel‐filtered platelets were incubated with the immobilized substrates at 37°C for 45 min (2 × 10^7^ platelets per well), and platelet spreading was visualized by confocal microscopy. Scale bar = 15 μm. (b) Percentage of platelets spread on BSA (negative control), fibrinogen (positive control), and recombinant PadA protein F2 fragments immobilized onto glass slides. Error bars represent ±SEM from three independent experiments (*n* = 3). NS = not statistically significant

Previously, we have suggested that the RGT and AGD motifs have little or no role in supporting platelet adhesion, but are involved in the transformation of the platelet biconcave disc through formation of filopodia and lamellipodia to a fully spread cell (Keane et al*.*, 2013). Accordingly, we tested the effect of NGR_AAA_ mutation on platelet spreading and found that for those platelets that adhered, spreading was unaffected (Figure 5). Taken collectively, these results suggest that NGR plays a major role in directing adhesion of platelets, while AGD and RGT promote spreading.

### 2.4 Functions of PadA and Hsa in binding fibronectin

We then utilized an affinity chromatography proteomics approach to determine if PadA interacted with specific host proteins present in human plasma. Purified PadA protein with a ×6 His C‐terminal tag (PadA_6His_) was linked to Ni‐NTA magnetic beads and incubated with plasma. The beads were collected, washed, and the interacting proteins were eluted, subjected to SDS‐PAGE, in‐gel digested with trypsin, and analysed by tandem mass spectrometry. Data analysis (see 4) identified Fn1 protein (fibronectin) (B7ZLE5_HUMAN) as the highest scoring protein to be pulled down (99% identity confidence/SEQUEST, 31.16% sequence coverage, 48 unique peptides) relative to plasma controls. Also, uncharacterized protein Q6GMX0_HUMAN was identified (47% sequence coverage, 1 unique peptide), recently annotated as anti‐polyhydroxybutyrate antibody Fv light chain, the significance of which is unclear. Fibronectin and Q6GMX0 peptides were low scoring or non‐detectable, respectively, in parallel plasma controls.

**Figure 6:**
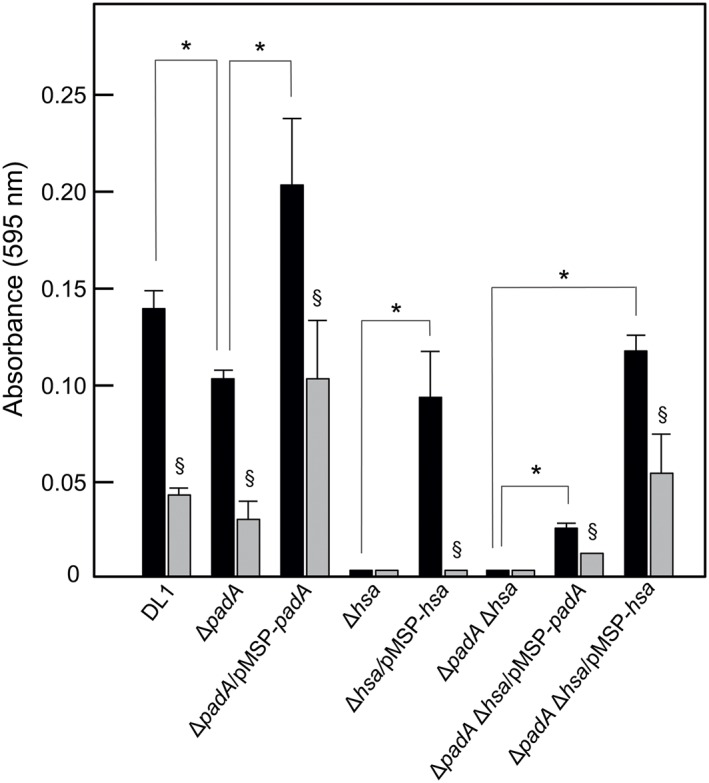
Adhesion of *S. gordonii* strains to human cellular fibronectin (cFn). Microtitre plate wells were coated with cFn (1 μg per well), blocked with BSA, and then incubated with streptococcal cells (5 × 10^7^ per well) for 2 hr at 37°C. Bacterial cells adhered (black columns) were quantified by staining with crystal violet as described in 4. Wells coated with cFn were also incubated with 0.001 U neuraminidase (sialidase) for 2 hr at 37°C, washed, blocked with BSA and then incubated with streptococcal cells (grey shaded columns). Expression of PadA or Hsa proteins by complemented strains was induced with 10 ng or 50 ng nisin ml^−1^. Error bars represent ±SEM from three independent experiments (*n* = 3). * *P* < 0.05 for comparisons indicated; § *P* < 0.05 for neuraminidase‐treated cFn versus untreated

Fibronectin is an extracellular matrix (ECM) protein often found at sites of endothelial cell damage to which platelets and bacteria are attracted. Plasma fibronectin (pFn) is a major component of the fibrin clot (early wound repair), while cellular fibronectin is involved in later repair events (To & Midwood, 2011). It is known that Hsa is involved in *S. gordonii* binding to pFn by recognition of sialylated regions on the molecule (Jakubovics et al*.*, 2009). Accordingly, we tested the Δ*padA* or Δ*hsa* mutants and complemented strains in adherence to pFn and to cFn. Levels of binding were slightly higher to cFn, but the overall adherence patterns of the strains were identical; therefore, we only present the data for binding to cFn. The Δ*padA* mutant was approximately 25% reduced in binding cFn, while the complemented strain was above wild type levels of binding (Figure 6). These adherence events were reduced by 50–60% when the cFn was desialylated (Figure 6). Deletion of *hsa* led to ablation of cFn binding, while complementation of the Δ*hsa* mutant led to part‐restoration of cFn‐binding activity (Figure 6). Complementation of the Δ*padA* Δ*hsa* mutant with *padA* led to a small increase in cFn binding, while complementation with *hsa* restored binding levels to just below wild type (Figure 6). We therefore conclude that Hsa is a major mediator of Fn binding under these conditions and that PadA plays a minor, but significant, role in the process. The fact that adhesion levels are only 50–60% reduced for desialylated cFn is consistent with the presence of PadA and other proteins that interact with non‐sialylated Fn (Jakubovics et al*.*, 2009).

### 2.5 Functions of PadA and Hsa in binding vitronectin

**Figure 7:**
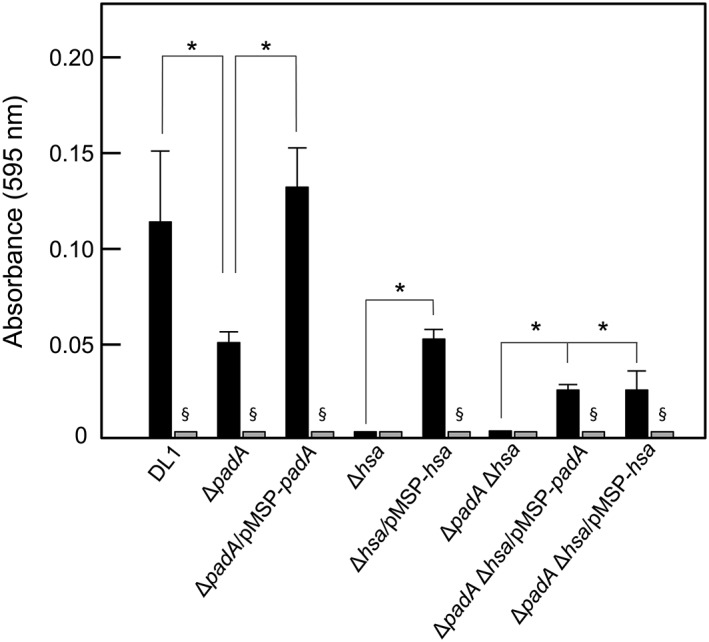
Adhesion of *S. gordonii* strains to human vitronectin (Vn). Microtitre plate wells were coated with Vn (0.05 μg per well), blocked with BSA, and then incubated with streptococcal cells (5 × 10^7^ per well) for 2 hr at 37°C. Bacterial cells adhered (black columns) were quantified by staining with crystal violet as described in 4. Wells coated with Vn were also incubated with 0.001 U neuraminidase (sialidase) for 2 hr at 37°C, washed, blocked with BSA, and then incubated with streptococcal cells (grey columns). Expression of PadA or Hsa proteins by complemented strains was induced with 10 ng or 50 ng nisin ml^−1^, respectively. Error bars represent ±SEM from three independent experiments (*n* = 3). * *P* < 0.05 for comparisons indicated; § *P* < 0.05 for neuraminidase‐treated Vn versus untreated

Vitronectin (Vn) is a component of the α‐granules of platelets as well as being found in ECM where it regulates proteolysis, promotes cell adhesion and spreading, and modulates the activities of components of the complement pathway (Singh, Su, & Riesbeck, 2010). Bacteria coated with Vn evade complement assault, and Vn cross‐links bacteria and host cells, thence triggering host cell signalling and cytoskeletal remodelling (Singh et al., 2010). Accordingly, we also determined the role of PadA and Hsa in binding to Vn. Deletion of *padA* resulted in 60% reduction in Vn‐binding levels (Figure 7) while adherence was fully restored by complementation with *padA* (Figure 7). Deletion of *hsa* abrogated binding of mutant cells to Vn, and complementation with ectopic *hsa* restored binding to about 45% of wild type levels. Interestingly, complementation of Δ*padA* Δ*hsa* with either *padA* or *hsa* alone did not complement the Vn‐binding phenotype. These results strongly indicate that Hsa requires the presence of functional PadA in order to efficiently bind Vn. Lastly, adherence of streptococcal cells to Vn was ablated by sialidase (neuraminidase) treatment of Vn, implying that Vn adherence is sialylation dependent (Figure 7).

### 2.6 Functions of PadA and Hsa in adherence to salivary glycoproteins and in biofilm formation

**Figure 8:**
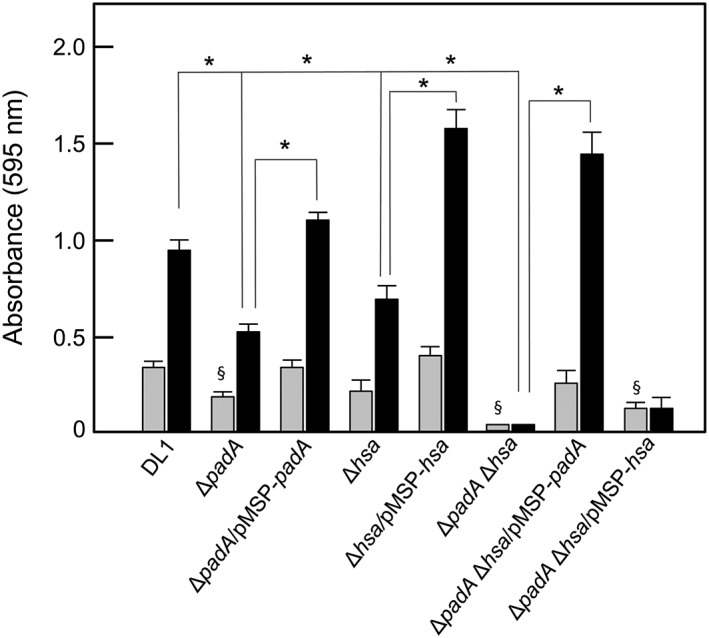
*S. gordonii* strains adherence to salivary pellicle and biofilm formation. Cover slips were coated with salivary pellicle and incubated with streptococcal cells (5 × 10^7^ per well) for 2 hr at 37°C for adherence (grey columns) or in YPT‐Glc medium for 16 hr at 37°C for biofilm formation (black columns). Bacterial cells adhered, and biofilm biomass values were quantified by staining with crystal violet as described in 4. Expression of PadA or Hsa proteins by complemented strains was induced with 10 ng or 50 ng nisin ml^−1^, respectively. Error bars represent ±SEM from three independent experiments (*n* = 3). * *P* < 0.05 for biofilm comparisons indicated; § *P* < 0.05 for significantly different adherence versus DL1

*S. gordonii* is normally found in the oral cavity and produces a spectrum of adhesins that interact with salivary components (Nobbs, Lamont, & Jenkinson, 2009). To test the effects of *padA* or *hsa* deletions on initial streptococcal adherence to salivary pellicle, bacteria were incubated with saliva‐coated glass cover slips, and levels of adhesion determined by crystal violet staining. We detected significant differences in adherence of the Δ*padA* and Δ*padA* Δ*hsa* mutants compared with wild type DL1 (Figure 8). Complementation with *padA* restored adherence levels while complementation with *hsa* did not (Figure 8).

In subsequent biofilm formation, the Δ*padA* mutant was 50% decreased in biomass compared to wild type, and complementation with *padA* restored biomass to slightly above wild type levels (Figure 8). Deletion of *hsa* resulted in similar effects, and complementation of the Δ*hsa* mutant was highly effective in enhancing biofilm formation. The Δ*padA* Δ*hsa* mutant was ablated in biofilm formation (Figure 8). Complementation with *pad*A restored biofilm formation, but complementation with *hsa* did not (Figure 8). These results suggest that PadA must be fully functional for Hsa to promote biofilm formation. However, it also appears that PadA alone can provide the necessary function for biofilm formation in the absence of Hsa (Figure 8).

### 2.7 PadA integrin‐recognition motifs are not involved in binding cFn, Vn, or salivary pellicle

**Figure 9:**
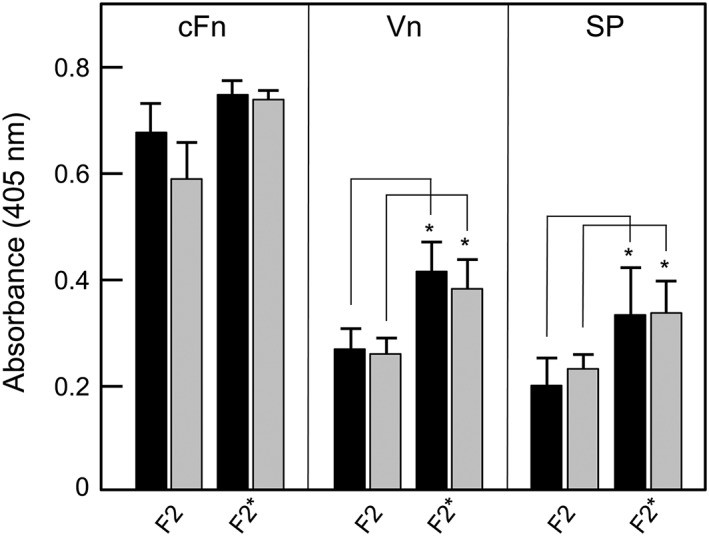
Adhesion of PadA‐F2 region fragments to cellular fibronectin (cFn), vitronectin (Vn), or salivary pellicle (SP). cFn, Vn, or saliva were immobilized onto microtitre plate wells and non‐specific binding sites were blocked with BSA (black columns). Wells coated with cFn, Vn, or salivary glycoproteins were also incubated with 0.001 U neuraminidase (sialidase) for 2 hr at 37°C, washed, and blocked with BSA (grey columns). Recombinant proteins (100 μg ml^−1^) were incubated with substrata for 2 hr at 37°C and amounts bound measured with anti‐tetra‐His mouse antibodies and HRP‐conjugated anti‐mouse IgG antibodies as described in 4. F2, unmodified fragment; F2*, fragment containing NGR, RGT, and AGD motifs all alanine‐substituted to AAA. Error bars represent ±SEM from three independent experiments (*n* = 3). * *P* < 0.05 compared with corresponding F2 values

Because the previous data strongly suggest that PadA is involved in binding ECM substrata and salivary pellicle, we tested the ability of the PadA‐F2 region to mediate these interactions, and the role of the three integrin‐recognition motifs. In binding assays of purified F2 fragments to immobilized substrata, it was found that levels of binding to cFn were higher than to Vn and pellicle (Figure 9). Substitution of all three integrin‐recognition motifs (RGT, AGD, and NGR) with AAA failed to significantly affect levels of adhesion of the PadA‐F2 fragment to Fn, but there was slightly elevated interaction with Vn and with salivary pellicle (Figure 9). Sialidase treatment of the substrata did not affect levels of PadA‐F2 binding compared to untreated substrata (Figure 9).

## 3 DISCUSSION

*S. gordonii* is a component of the normal microbiota of the human oral cavity and plays a pivotal role in the development of microbial communities on the tooth surfaces and gingival crevices (Jenkinson, 2011; Wright et al*.*, 2013). These communities provide a reservoir for bacteria to enter the circulation where they may interact with blood platelets and cause unwanted thrombus generation. This can lead to infective endocarditis, which is characterized by the formation of vegetations on the heart valves (Moreillon & Que, 2004). Significant advances in our understanding of how *S. gordonii*, and other mitis‐group oral bacteria, interact with platelets have been made in recent years.

In addition to the knowledge about *S. gordonii* Hsa and GspB proteins (Takahashi et al*.*, 2002; Xiong, Bensing, Bayer, Chambers, & Sullam, 2008), and how they are able to interact with platelet integrin GPIb (Takamatsu et al*.*, 2005), we have demonstrated that PadA surface protein can bind to platelets in a α_IIb_β_3_‐dependent manner (Petersen et al*.*, 2010) causing dense granule secretion and full platelet spreading (Keane et al*.*, 2010). The N‐terminal region of 1328 aa residues interacts with platelets (Keane et al*.*, 2013; Petersen et al*.*, 2010), and here, we show that the same region binds Vn, Fn, and salivary pellicle. The PadA C‐terminal region comprising 2,318 aa residues has no defined function at this stage. It is thought that this region containing aa residue repeat blocks (Figure 1) may act as a flexible stalk holding the N‐terminal binding‐region of the protein out into the environment. C‐terminal region repeat‐block regions of other *S. gordonii* surface proteins, such as CshA and Hsa, are reported to provide extended conformations (McNab et al*.*, 1999; Takahashi et al*.*, 2002) that may assist in capture of their ligands even under conditions of shear or flow (Kerrigan et al*.*, 2007).

In the first part of the present study, we focused on the roles of three integrin‐like recognition motifs within the N‐terminal PadA‐F2 fragment (657 aa residues) in the interactions of PadA with platelets. We showed previously that neither RGT_(416–418)_ nor AGD_(485–487)_ were necessary for supporting static or shear‐induced platelet adhesion, but both motifs contributed to platelet spreading (Keane et al., 2013). This is in keeping with the data suggesting that Hsa is essential for platelet capture under shear and thus responsible for platelet rolling (Bensing et al*.*, 2004; Kerrigan et al*.*, 2007). Firm adhesion is then complete when platelet integrin α_IIb_β_3_ interacts with PadA (Petersen et al*.*, 2010). Recent evidence indicates that another motif, NGR, within fibrinogen is responsible for interaction with α_IIb_β_3_ and triggering platelet activation (Moriarty et al*.*, 2015). In light of this discovery, we investigated the role of an identical motif NGR_(214–216)_ within the N‐terminal region of PadA to direct interactions with platelets. In our experiments with platelets that were specifically in resting state, we found that the PadA‐F2 fragment bound these only weakly (Figure 4a). However, the immobilized PadA‐F2 region bound TRAP‐activated platelets, so this would be in keeping with the notion that PadA preferentially interacts with α_IIb_β_3_ that is in an activated complex with GPIb following Hsa‐binding. Confirming our previous studies, we showed that RGT and AGD were not essential for PadA‐F2 binding to TRAP‐activated platelets. However, binding of the PadA‐F2 fragment to activated platelets required the combined activities of NGR, RGT, and AGD, because alanine‐substitutions of all of these motifs ablated adhesion (Figure 4b). Moreover, we could not demonstrate that the NGR motif alone was necessary for full platelet spreading, while RGT and AGD motifs clearly contribute to the full spreading process (Keane et al*.*, 2013). In summary, we are nearing the situation in which we could potentially target these three motifs within PadA as a means to controlling unwanted platelet activation by circulating *S. gordonii*, and by other oral streptococci that express PadA‐like proteins.

In this study, we have also utilized gene knockouts and respective complemented strains of *S. gordonii* in order to dissect the relative functions of PadA and Hsa in host tissue interactions. Our results have uncovered some unexpected dependencies of the two proteins on each other for their adhesive functions. Platelet adhesion depends upon the dual functioning of Hsa and PadA, as previously interpreted, but knock‐in of PadA to a Δ*padA* Δ*hsa* double mutant does not restore adhesion, only knock‐in of Hsa part‐restores adhesion. This appears to support evidence that PadA contributes to adhesion only when platelets have first been engaged with Hsa. The fact that knock‐in of Hsa does not fully restore platelet adhesion shows the requirement of PadA for complete engagement (Figure 3).

Our experiments with *S. gordonii* mutants also demonstrate that PadA and Hsa are involved in binding Fn and Vn. Binding of *S. gordonii* to cFn is in part sensitive to sialidase treatment of cFn, and this is consistent with the known activity of Hsa in Fn‐binding by *S. gordonii* (Jakubovics et al*.*, 2009). It is possible that regions outside the Siglec‐like sialic acid‐binding domain within the BR (binding region) of Hsa (Pyburn et al., 2011) interact with Fn. For example, the BRs of Streptococcus agalactiae serine‐rich repeat proteins Srr1 and Srr2 have been shown to bind fibrinogen (Seo et al., 2013). Another *S. gordonii* surface protein that interacts with Fn is CshA (McNab et al*.*, 1999). Neither CshA nor PadA contain primary sequences or motifs that have been associated with Fn recognition by other Gram‐positive bacterial cell surface proteins (Henderson, Nair, Pallas, & Williams, 2011). The mechanism of cFn binding by PadA is currently under investigation. In summary, Hsa is a major component of the *S. gordonii* cell surface that binds sialylated Fn. PadA also binds cFn, but this is less effective in the absence of Hsa (Figure 6) suggesting that, like for platelets, Hsa binding sialic acid residues promotes PadA function.

The finding that Δ*padA* and Δ*hsa* mutants of *S. gordonii* are also deficient in binding to Vn may have significance in the overall processes of platelet binding and activation, and in immune evasion. Vn is present in plasma, ECM and in platelet α‐granules. Blood platelets secrete multimeric Vn following activation, although about 50% remains platelet bound (Parker, Stone, White, & Seinshaw, 1989). Vn forms complexes with plasminogen activator inhibitor‐1 (PAI‐1) and thus behaves as a physiological inhibitor of active thrombin. Conversely, Vn also appears to stabilize the thrombus and, so, plays a dual role in mediating platelet adhesion (Thiagarajan & Kelly, 1988) and aggregation (Reheman et al*.*, 2005). Vn carries binding sites for heparin, PAI‐1, integrins, collagen and plasminogen (Ekmekçi & Ekmekçi, 2006) and also binds α_IIb_β_3_. Thus, *S. gordonii* might be able to interact with platelets via Vn, which may be bound to β_3_ integrins or to surface‐associated collagen. Furthermore, bacteria coated with Vn are able to evade the membrane attack complex (MAC) by blocking complement components (C5b‐7 complex and C9) and conferring serum resistance (Singh et al*.*, 2010). This of course would be a crucially important property for augmenting systemic survival of bacteria. Our data suggest that binding of streptococcal cells to Vn is dependent on glycan moieties, in particular sialic acid, because sialidase treatment of Vn led to ablation of streptococcal cell adhesion. The major *N*‐linked oligosaccharides of Vn consist of the *N*‐acetyllactosamine type, with a major proportion of them sialylated (Ogawa et al*.*, 1995). Our adherence data for knock‐out and complemented mutants strongly suggest that both PadA and Hsa are involved in Vn recognition, since complementation of Δ*padA* Δ*hsa* double knock‐out mutants with either PadA or Hsa was not sufficient to restore Vn binding. This again provides evidence for concerted activities of PadA and Hsa. Overall, PadA would seem therefore to be a versatile molecule with a range of properties that would enable it to modulate thrombogenesis, accelerate the thrombotic response, facilitate the incorporation of bacterial cells into vegetations and into hemostatic plaques (Brown, Lundgren, Nordt, & Fujii, 1994) and help *S. gordonii* resist complement‐mediated lysis by binding Vn.

Experiments investigating adherence to salivary pellicle and biofilm formation set out to determine if there were roles for the PadA and Hsa proteins in oral cavity colonization outside of pathogenesis within the circulatory system. It is already known that Hsa and GspB bind salivary proteins (Takamatsu, Bensing, Prakobphol, Fisher, & Sullam, 2006), and that Hsa is required for intergeneric coaggregation with *Veillonella* species (Zhou, Liu, Li, Takahaski, & Qi, 2015). However, this is not sialic acid‐dependent, providing further evidence for the presence of additional binding sites within the BR of Hsa. The ability of *S. gordonii* to adhere to pellicle is antecedent to biofilm formation. Both PadA and Hsa appear to contribute to biofilm formation and complementation of Δ*padA* or Δ*hsa* single knock‐out mutants restores the ability to form biofilms in each case. However, only complementation of the Δ*padA* Δ*hsa* mutant with PadA restored biofilm formation, not complementation with Hsa. We conclude with an important interpretation that the role of Hsa in biofilm formation is only effective in the presence of PadA. Clearly, therefore, PadA has the ability to mediate *S. gordonii* binding to salivary pellicle in the absence of sialic acid receptors (Figure 9), and to promote cell–cell interactions that also do not involve sialic acid receptors and result in biofilm formation. This could mean that biofilm formation is a two‐step process, and that PadA provides the first step (as opposed to the secondary step in binding platelets). Alternatively, Hsa and PadA may independently contribute to biofilm formation but Hsa is not functionally expressed without the presence of PadA. This might suggest that a complex is formed between PadA and Hsa on the *S. gordonii* cell surface, and this possibility is currently under investigation.

**Figure 10:**
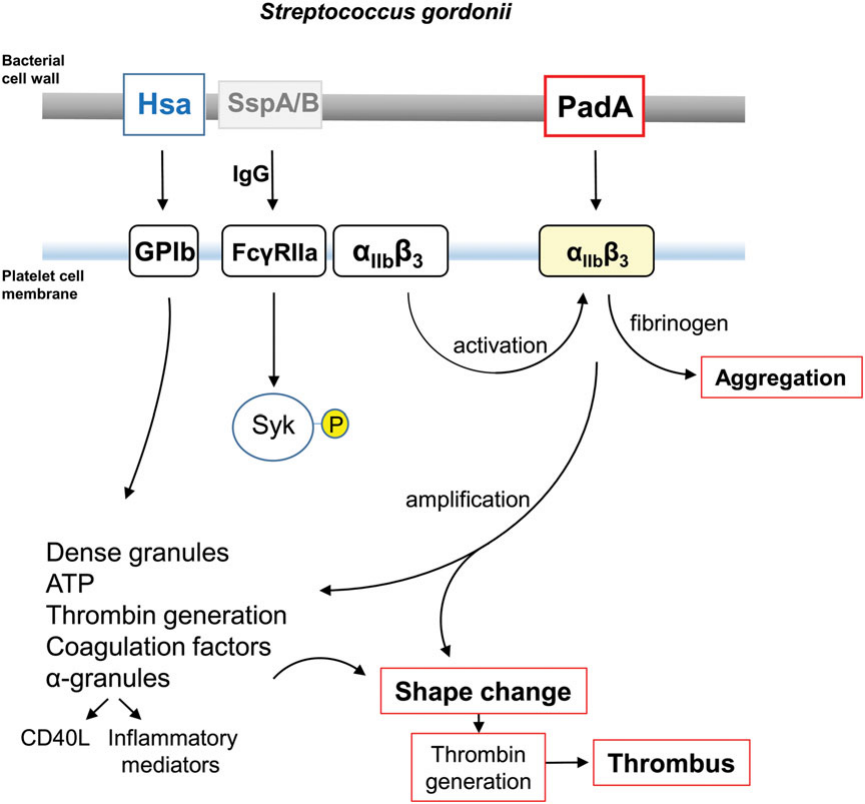
Diagrammatic representation of some of the processes involved in platelet activation by *S. gordonii*. Cell wall‐anchored proteins Hsa and PadA interact with platelet membrane integrins GPIb and α_IIb_β_3_ (GPIIbIIIa). Hsa captures platelets under flow (rolling) by binding GPIb, and possibly also α_IIb_β_3_, and activates signalling cascades including FcγRIIa phosphorylation, leading to dense granule release (see Arman et al., 2014). PadA binds activated α_IIb_β_3_, thus amplifying signals leading to shape change, thrombin production, coagulation, and thrombus formation. Platelet activation by *S. gordonii* can occur in the absence of specific IgG. However, with IgG present, there is evidence for activation (phosphorylation) of spleen tyrosine kinase (Syk‐P) through FcγRIIa. Conserved streptococcal surface protein antigens such as antigen I/II proteins (e.g., SspA/B) may also be involved in the overall process (Kerrigan et al., 2007). Physiologically, collagen activates Syk through GPVI, which is closely associated with FcγRIIa (not shown). Fibrinogen engages GPIIbIIIa (α_IIb_β_3_), which also associates with FcγRIIa. CD40L (otherwise known as CD154) is up‐regulated in the platelet cell membrane and binds CD40^+^ cells such as endothelial cells and neutrophils, while soluble (released) CD40L further activates platelets

In conclusion, PadA is a multi‐domain adhesin that interacts with activated platelets via α_IIb_β_3_ facilitating firm adhesion, granule release, and full platelet spreading (see Figure 10). These properties depend upon the presence of NGR, RGT, and AGD integrin‐like recognition motifs within the N‐terminal F2 fragment of 657 aa residues. These motifs are not involved in PadA‐F2 fragment binding to cFn, Vn, or salivary pellicle, suggesting that they are more critical to platelet interactions, and that the alanine substitutions did not significantly affect the general adhesion properties of the F2 region. PadA functions in concert with Hsa, mediating firm adhesion of platelets following capture of platelets by Hsa via GPIb, and firm adhesion to cFn. PadA and Hsa also act cooperatively in mediating binding of bacteria to Vn, salivary pellicle, and biofilm formation. In the latter phenotype, Hsa is clearly reliant on PadA expression to mediate its function. Taken collectively, these results strongly suggest that in *S. gordonii* at least two cell wall‐anchored proteins work in concert to mediate host‐interactive processes relevant to both bacterial colonization and pathogenesis.

## 4 EXPERIMENTAL PROCEDURES

### 4.1 Bacterial growth conditions

*Streptococcus gordonii* DL1‐Challis, isogenic mutants UB2723 Δ*padA*::*aad9*, UB2029 *Δhsa*::*aphA3*, UB2773 *ΔpadA*::*aad9 Δhsa*::*aphA3*, and mutant strains complemented with pMSP‐*padA* or pMSP‐*hsa*, were routinely grown on BHY agar (37 g l^−1^ Brain Heart Infusion, 5 g l^−1^ Yeast Extract, 15 g l^−1^ agar) or in BHY broth, at 37°C under CO_2_‐enriched conditions (candle jar). Less complex TY medium (5 g l^−1^ Bacto‐tryptone, 4 g l^−1^ yeast extract, 4 g l^−1^ K_2_HPO_4_, adjusted to pH 7.5 with HCl) containing 0.5% glucose (TY‐Glc) was employed for protein purification from *S. gordonii* UB2870 Δ*padA*/pMSP‐*padA*_*6His*_. Protein expression from pMSP was under control of a nisin‐inducible (10–50 ng ml^−1^) promoter (Bryan, Bae, Kleerebezem, & Dunny, 2000; Hirt, Erlandsen, & Dunny, 2000). Escherichia coli strains DH5α, JM109, XL1‐Blue, Stellar® and BL21 for plasmid preparation and protein expression were grown on LB agar or in LB broth at 37°C. Antibiotics were incorporated where necessary for streptococci [5 μg erythromycin (Em) ml^−1^; 100 μg spectinomycin (Sp) ml^−1^; 250 μg kanamycin (Kn) ml^−1^] or E. coli [100 μg ampicillin (Ap) ml^−1^; 250 μg Em ml^−1^].

### 4.2 Generation of *S. gordonii* gene knockout strains

Plasmids or PCR amplimers were purified using QIAquick Spin Miniprep or PCR Purification kits respectively (Qiagen, Manchester, UK). Oligonucleotides (Table 1) were synthesised by MWG Eurofins (Wolverhampton, UK). Chromosomal DNA was extracted from mutanolysin‐treated *S. gordonii* DL1 cells, as described previously (Jenkinson, 1987). DNA restriction and modification enzymes were used under the conditions specified by the manufacturer (New England Biolabs, Hitchin, Herts, UK).

The *padA* gene of *S. gordonii* DL1 was deleted by allelic exchange with the Sp resistance determinant *aad9*. PCR amplification using Expand Long PCR System (Roche, Burgess Hill, West Sussex, UK) with primers padA.F1/padA.R1 and padA.F2/padA.R2 and *S. gordonii* DL1 chromosomal DNA template generated two fragments comprising the flanking sequences of the *padA* gene (676 bp, 518 bp) with a unique NarI site at their 3′ and 5′ ends, respectively. These were stitched together in a second round of PCR using primers padA.F1/padA.R2 generating a 1155‐bp amplimer with an internal unique NarI site. The fragment was subsequently cloned into pGEM‐T Easy to generate construct pGEM‐*padA*flank and transformed into chemically‐induced competent cells (Hanahan, 1983) of Escherichia coli XL1‐Blue. The *aad9* gene was PCR amplified from plasmid pFW5 (Podbielski, Spellerberg, Woischnik, Pohl, & Lútticken, 1996) using primers aad9F.NarI/aad9R.NarI and then sub‐cloned into the NarI site of pGEM‐*padA*flank to generate construct pGEM‐*padA*flank::*aad9*. PCR amplification from pGEM‐*padA*flank::*aad9* with primers padA.F1/padA.R2 generated amplimer *padA*flank::*aad9,* which was transformed into *S. gordonii* DL1 as described by Haisman and Jenkinson (1991) to produce the *padA* gene knockout mutant (designated UB2723). Confirmation of successful mutagenesis was obtained by sequencing the PCR product amplified from the chromosomal locus with primer pair padA.F1/padA.R2. Absence of PadA protein from the cell wall of the mutant was verified by Western immunoblotting using anti‐PadA antibodies (Petersen et al*.*, 2010).

To generate a *S. gordonii* DL1 Δ*padA* Δ*hsa* double‐knockout mutant, *S. gordonii* UB2029 Δ*hsa* *::aphA3*, a reisolate of UB1545 (Jakubovics et al*.*, 2005), was used as recipient for allelic replacement of the *padA* gene with *aad9*. Primers padA.F1/padA.R2 were utilized to amplify with PrimeSTAR Max polymerase (Takara Biotechnology, Shiga, Japan) a 2‐kb fragment from UB2723 chromosomal DNA. The amplimer was purified and transformed into *S. gordonii* UB2029 with selection for Kn^R^ and Sp^R^, and colonies were screened by PCR using primers aad9.seqF/padA.R2 to detect the *aad9* cassette. Several transformants were checked by sequencing PCR products generated from across the loci and a representative transformant was purifed and designated UB2773.

### 4.3 Generation of *S. gordonii* complemented mutant strains

To complement the *S. gordonii* UB2723 Δ*padA* mutant, the entire *padA* coding sequence was cloned into pMSP, a derivative of pMSP7517 (Hirt et al*.*, 2000) in which the *prgB* gene had been replaced by DNA encoding three alanine residues, together with the transcriptional terminator of the *padA* operon. In brief, a 227‐bp region downstream of SGO_RS09805 incorporating the *padA* operon transcriptional terminator was amplified from *S. gordonii* DL1 chromosomal DNA using PrimeSTAR® GXL polymerase (Takara Biotechnology, Shiga, Japan) with primers Terminator.XhoF/Terminator.XhoR. The amplimer was cloned using In‐Fusion HD Cloning Kit (Clontech, Oxford, UK) into vector pMSP, which had been linearized by digestion with XhoI. The primers were designed such that only the XhoI site at the 5′ end of the transcriptional terminator would be retained upon cloning. This construct (pMSP‐term) was transformed into E. coli K12 and confirmed by sequencing. The *padA* gene (10,967 bp) was then amplified using primers padA.pMSPF/padA.pMSPR and cloned via XhoI sites at its termini into pMSP‐term that had been similarly digested. The resulting construct (pMSP‐*padA*) was transformed into E. coli K12 and confirmed by sequencing. The construct was then transformed into *S. gordonii* UB2723 Δ*padA* and into UB2773 Δ*padA* Δ*hsa* to generate complemented mutants. PadA expression was under control of the nisin‐inducible promoter present in pMSP, and so following nisin‐induction (10–50 ng ml^−1^) PadA expression by the complemented strains was confirmed by Western immunoblot of cell wall protein extracts and whole cell FACS analyses, with anti‐PadA antibodies (Figure 2).

To purify PadA from *S. gordonii*, plasmid pMSP‐*padA* was used as template in inverse PCR with 5′ phosphorylated primers His.padAR 5′ATGATGATGTGCTCCGTCTTTAATAGATG and His.padAF CACCACCACTAACTCGAGGAATTAGGTTG to substitute the sequence encoding the PadA wall anchorage motif (LPKTG) with a sequence encoding ×6 His (underlined in primers), followed by a stop codon. Amplicons were ligated following removal of original template by DpnI digestion, and resulting plasmids were transformed into E. coli JM109. Several plasmids were confirmed by sequencing, and then transformed into *S. gordonii* UB2723 Δ*padA.* A representative strain was selected in which *padA* gene expression was induced with nisin (50 ng ml^−1^), and PadA protein, in the absence of cell wall anchorage motif, was secreted into the growth medium (Figure S1).

Generation of plasmid pMSP*‐hsa* carrying the complete coding sequence of *hsa* and *S. gordonii* strain Δ*hsa*/pMSP‐*hsa* (UB1746) have been described previously (Jakubovics et al*.*, 2009). To make a complement in *S. gordonii* UB2773 Δ*hsa* Δ*padA* double mutant, pMSP‐*hsa* was transformed into this strain to generate *S. gordonii* UB2777 Δ*hsa* Δ*padA*/pMSP‐*hsa.* The *S. gordonii* pMSP‐*hsa* complemented strains were induced with nisin (10–50 ng ml^−1^) and subjected to lectin immunoblotting with sWGA to confirm Hsa expression (Jakubovics et al*.*, 2009; Figure 2).

### 4.4 Site‐directed mutagenesis and protein expression

Site‐directed mutagenesis of two potential integrin‐recognition sites in the F2 N‐terminal region of PadA have been previously described (Keane et al*.*, 2013). Briefly, alanine‐substitution mutagenesis was performed by the use of mutagenic primers containing base mismatches in inverse PCR amplification of pET46‐*F2,* containing the PadA‐F2 domain coding sequence to generate pET46‐*F2* NGR_AAA_ *,* pET46‐*F2* RGT_AAA_ or pET46‐*F2* AGD_AAA_ *.* In addition to the base mismatches corresponding to the alanine substitutions, a new engineered restriction site was introduced into each plasmid, enabling restriction digest to generate compatible ends for self‐ligation to reform a circular plasmid.

A modified alanine substitution method was employed to generate double and triple motif substitutions. In summary, this technique avoided the need for restriction digests to prepare complementary ends and instead made use of 5′ phosphate groups on the primers to enable plasmid self‐ligation. pET46‐*F2* AGD_AAA_ was extracted and purified from E. coli JM109 and used as template DNA for inverse PCR amplification using primers JH5F/JH6R. These primers incorporated 5′phosphate groups and base mismatches to generate both the alanine substitutions and an engineered AgeI site to facilitate screening by restriction digest analysis. Phusion DNA Polymerase (Life Technologies, Paisley, UK) was used in a two‐step protocol to generate the desired amplimer of 7200 bp. This was treated with DpnI for 1 hr at 37°C to remove template DNA and subsequently purified. The purified DNA was self‐ligated using T4 DNA ligase for 16 hr at 16°C and subsequently transformed into competent E. coli JM109 cells. Transformants were selected for Ap^R^, and colonies were screened for plasmids that were cut with AgeI, indicating the formation of pET46‐*F2* RGT_AAA_AGD_AAA_, and this was confirmed by sequencing. Purified plasmid was then transformed into competent E. coli BL21 cells for recombinant protein production (see below).

Generation of a pET46‐*F2* NGR_AAA_RGT_AAA_AGD_AAA_ plasmid construct was achieved using a similar alanine substitution technique to that used to generate the pET46‐*F2* RGT_AAA_AGD_AAA_ construct*.* Briefly, the template plasmid pET46‐*F2* RGT_AAA_AGD_AAA_ was extracted and purified from E. coli JM109. 5′‐phosphate‐modified JH19F and JH20R primers, which incorporated an engineered NotI restriction site, were used in Phusion PCR to generate the desired amplimer (7200 _BP_), which was subsequently treated with DpnI, self‐ligated and transformed into competent E. coli JM109 cells. Plasmids from transformant cells were extracted, purified, and analysed by NotI restriction digest. Plasmids that gave the correct NotI restriction digest pattern were likely to be the desired constructs. This was confirmed by sequencing, before transformation of individual plasmids into competent E. coli BL21 cells.

### 4.5 Protein expression and purification

Recombinant protein expression was induced in E. coli BL21 cultures at OD_600_ 0.5–0.7 by adding IPTG at a final concentration of 1 mM, and continuing incubation for 4 hr at 37°C with shaking at 220 rpm. Cells were harvested by centrifugation (5000 × *g*, 10 min), and the pellet was lysed in 2 ml Buffer B (8 M urea, 0.1 M NaH_2_PO_4_, 0.01 M Tris–HCl, pH 7.0) supplemented with 0.1 M phenylmethanesulfonylfluoride (PMSF) at room temperature for 4 hr. Cell debris was removed by centrifugation (12,000 × *g*, 15 min). Ni‐NTA agarose (Qiagen; 300 μl) was added to 2 ml lysis supernatant, mixed gently at room temperature for 25 min, and then transferred to a polypropylene column (Qiagen). The column was washed with 4 ml Buffer B (supplemented with 20 mM imidazole) and then twice with Buffer C (1 M NaCl, 10% EtOH, 2% Tween‐20) supplemented with 20 mM imidazole. Protein was eluted six times with Buffer D (Buffer B, supplemented with 100 mM imidazole). All of the washes and eluted fractions were collected and analysed by SDS‐PAGE. Fractions containing recombinant proteins were pooled and dialysed against dH_2_O supplemented with protease inhibitor cocktail (Sigma). Dialysed protein solutions were freeze‐dried, suspended in dH_2_O, and the protein concentrations determined using the Bradford assay (BioRad Laboratories, Hemel Hempstead, Herts, UK).

### 4.6 PadA_6His_ expression and purification from *S. gordonii*

*S. gordonii* UB2870 Δ*padA*/pMSP‐*padA*_*6His*_ cultures were grown in TY‐Glc medium at 37°C with Sp, Em and 100 ng ml^−1^ nisin, to early stationary phase. The cultures were centrifuged (12,000 × g, 4°C, 20 min), the supernatants were collected, protease inhibitor cocktail (Sigma) added, and then passed through a 0.45 μm filter. The fluid was concentrated to 4 ml, using a centrifugal filter unit, and centrifuged (12,000 × g, 4°C, 10 min) to remove particulate matter. The fluid was then dialysed into Buffer A (50 mM Tris, 200 mM NaCl, 10% glycerol, 0.1% Triton X‐100, pH 7.5) for 16 hr at 4°C. A HiTrap Ni^2^^+^ affinity column (GE Healthcare, Cardiff, UK) was pre‐equilibrated in Buffer A, the protein sample was loaded onto the column, and non‐specifically bound proteins were removed by washing the column first with Buffer A (5 ml) and then with Buffer B (50 mM Tris, 200 mM NaCl, 10% glycerol, 0.1% Triton X‐100, 20 mM imidazole, pH 7.5). The PadA_6His_ protein was eluted with 15 ml Buffer C (50 mM Tris, 200 mM NaCl, 10% glycerol, 0.1% Triton X‐100, 500 mM imidazole, pH 7.5) and collected in 3 ml fractions. These were analysed by SDS‐PAGE and fractions containing pure PadA_6His_ were pooled and dialysed into Buffer A for 16 hr at 4°C. Identity and purity were confirmed by liquid chromatography‐mass spectrometry (LC–MS) analysis (University of Bristol Proteomics Facility).

### 4.7 Pull‐down assay

Dynabeads® His‐Tag Isolation and Pulldown kit (Life Technologies) was used for PadA‐serum pull‐down assays according to the manufacturer's recommended instructions. PadA_6His_ (4 mg ml^−1^) was diluted in Binding/Wash buffer and incubated with mixed pooled human plasma (TCS Biosciences, Botolph Claydon, Bucks, UK) diluted 1:5 in Pull‐down buffer. Elution steps were performed according to the manufacturer's instructions.

### 4.8 Mass spectrometry sample preparation

To prepare the eluted samples from the pull‐down assays for MS, the samples were subjected to SDS‐PAGE until the dye‐front had moved 1.5 cm into the gel. The gel was stained with Coomassie blue, destained, and a single gel slice encompassing proteins below 350 kDa was excised. The slice was subjected to in‐gel tryptic digestion with a ProGest automated digestion unit (Digilab UK). The resulting peptides were fractionated with a Dionex Ultimate 3000 nanoHPLC system in line with an LTQ‐Orbitrap Velos mass spectrometer (Thermo Scientific) controlled by Xcalibur 2.1 software (Thermo Scientific). The Orbitrap was set to analyse the survey scans at 60,000 resolution (at m/z 400) in the mass range m/z 300 to 2,000 and the top twenty multiply‐charged ions in each duty cycle were selected for MS/MS in the LTQ linear ion trap.

### 4.9 Liquid chromatography‐mass spectrometry data analysis

The raw data files were processed and quantified using Proteome Discoverer software v1.2 (Thermo Scientific) and searched against the UniProt/SwissProt *S. gordonii* (strain Challis/ATCC 35105/CH1/DL1/V288) database (2,056 entries) and the UniProt/SwissProt Homo sapiens database (70,625 entries) using SEQUEST (Ver. 28 Rev. 13) algorithm for the pull‐down elution samples. Peptide precursor mass tolerance was set at 10 ppm, and MS/MS tolerance was set at 0.8 Da. Search criteria included carbamidomethylation of cysteine (+57.0214) as a fixed modification and oxidation of methionine (+15.9949) as a variable modification. Searches were performed with full tryptic digestion, and a maximum of one missed cleavage was allowed. The reverse database search option was enabled, and all peptide data were filtered to satisfy false discovery rate (FDR) of 5%. The Proteome Discoverer software generates a reverse “decoy” database from the same protein database, and any peptides passing the initial filtering parameters that were derived from this decoy database are defined as false positive identifications. The minimum cross‐correlation factor (Xcorr) filter was readjusted for each individual charge state separately to optimally meet the predetermined target FDR of 5% based on the number of random false positive matches from the reverse decoy database. Thus, each data set has its own passing parameters.

### 4.10 Platelet adherence assay

Whole blood was collected from six donors and added to 1.5 ml acid citrate dextrose per 10 ml blood collected. The donors were healthy subjects who had abstained from taking any non‐steroidal anti‐inflammatory drugs (NSAIDs) in the previous 10 days. Informed consent was obtained from all subjects, and the study was approved by the Royal College of Surgeons in Ireland Ethics Committee (REC679b). Blood was centrifuged (150 × *g*, 10 min), and the upper platelet‐containing fraction was carefully removed to a fresh tube. The platelet‐rich plasma (PRP; collected in acid‐citrate dextrose, ACD) was adjusted to pH 6.5 with ACD. Apyrase (1 U ml^−1^), and prostaglandin‐E1 (Sigma Aldrich; 2 μM) were added to the PRP, and the sample was centrifuged (650 × *g*, 10 min, 20°C). The upper layer containing platelet‐poor plasma was removed, and the platelet pellet was suspended in 2 ml modified HEPES‐Tyrode buffer (JNL; 6 mM dextrose, 130 mM NaCl, 9 mM NaHCO_3_, 10 mM Na citrate, 10 mM Tris base, 3 mM KCl, 0.8 mM KH_2_PO_4_, 0.9 mM MgCl_2_, pH 7.4). The platelet suspension was then applied to a chromatography column containing 5 ml packed Sepharose 2B‐300, previously equilibrated with JNL buffer. The platelet concentration was determined using a Sysmex‐100 particle counter (Sysmex, Kobe, Japan).

Static platelet adhesion assay was performed as described previously (Kerrigan et al*.*, 2002). Briefly, 96‐well plates were coated with bacteria (1 × 10^9^ cells ml^−1^), recombinant protein fragments, fibrinogen, or BSA (each at 100 μg ml^−1^). Gel‐filtered platelets (4 × 10^8^ cells ml^−1^) were added to each well and incubated for 45 min at 37°C. Following a gentle wash to remove unattached platelets, lysis buffer containing *p*‐nitrophenol phosphate was added to the wells and incubated for 20 min at 37°C. The resultant absorbance at 405 nm (*A*_405_) was a measure of acid phosphatase activity, proportional to numbers of platelets bound. Where appropriate, platelets were activated with 20 μM TRAP sequence SFLLRN (Sigma Aldrich, Wexford, Ireland).

### 4.11 Platelet spreading assay

Poly‐L‐lysine‐coated glass slides were coated with PadA protein fragment (100 μg ml^−1^) for 16 hr at 4°C. Slides were then blocked with 1% BSA; washed and gel‐filtered platelets (5 × 10^6^ platelets ml^−1^) were allowed to spread on the PadA fragments for 45 min at 37°C. The slides were washed, the platelets were fixed and permeabilized, and then the samples were stained with Alexa 546 phalloidin and examined by confocal microscopy as described in detail elsewhere (Keane et al., 2013). The percentage of platelets spread was calculated from five areas, selected randomly, containing 200 or more total cells.

### 4.12 Bacterial cell adhesion assays

Extracellular matrix proteins in coating buffer (20 mM Na_2_CO_3_, 2 mM NaHCO_3_, pH 9.3) were added to the wells of a high‐binding 96‐well plate (Immulon 2HB) and incubated for 16 hr at 4°C. In some experiments, immobilized substrata were then desialylated with 0.001 U neuraminidase from Clostridium perfringens (Sigma) in 0.1 M sodium acetate buffer (pH 5.0) containing 2 mM CaCl_2_, for 2 hr at 37°C. Wells were washed once in TBSC (10 mM Tris–HCl pH 7.6, 0.15 M NaCl, 5 mM CaCl_2_), and non‐specific binding sites blocked with 3% BSA in TBSC containing 0.05% Tween‐20 (TBST) for 1 hr at 37°C. Bacterial cultures were grown for 16 hr at 37°C, and cells were harvested by centrifugation (5000 × *g*, 7 min). Cells were washed once in TBSC, adjusted to OD_600_ 0.5, and portions (0.1 ml) incubated with the immobilized proteins at 37°C for 2 hr. The suspensions were then removed, and the wells were washed twice in TBS. Adherent cells were fixed with 25% formaldehyde for 30 min at room temperature. Wells were washed twice in TBS, 0.5% crystal violet was added, and the plates were incubated for 2 min at room temperature. Wells were washed three times in TBS, 10% acetic acid (0.1 ml) was added and after 5 min, levels of adhesion were quantified by measuring absorbance at 595 nm (*A*_595_). Previous work has shown that bacterial cell numbers are proportional to A_595_ over the range employed in the assays (Jakubovics et al*.*, 2005).

### 4.13 Salivary pellicle adhesion assay

Saliva was collected from at least six healthy adult human subjects, who provided written informed consent (approved by the National Research Ethics Committee South Central Oxford C. 165 # 08/H0606/87 + 5). Exclusion criteria were: antimicrobial medication within 7 days previously, continuous medication, gross dental caries, or unstable periodontal disease. Samples were pooled, mixed with 0.25 M dithiothreitol on ice for 10 min and clarified by centrifugation (8000 × *g*, 10 min). The supernatant was diluted to 10% with sterile water, filter sterilized (0.22 μm pore membrane) and aliquots were stored at −20°C.

Saliva‐coated cover slips were prepared by placing sterile 19‐mm‐diameter cover slips (Menzel‐Glaser, Braunschweig, Germany) into 12‐well plates (Greiner) and adding 10% saliva (1 ml) to each well. The cover slips were incubated at 4°C for 16 hr, washed with PBS, and transferred to a fresh 12‐well plate. Bacterial cell suspension (OD_600_ 0.5) was added to each well as described above, incubated at 37°C for 2 hr, and aspirated from the wells, and the cover slips were washed twice in TBSC. Adherent cells were fixed and stained with crystal violet, and levels of adhesion were quantified by *A*_595_ measurement.

### 4.14 Biofilm formation

Bacterial cultures grown for 16 hr (10 ml) were harvested by centrifugation (5000 × *g*, 7 min), washed in modified C (mC) medium (0.25% Difco Proteose Peptone #2, 0.75% yeast extract, 10 mM K_2_HPO_4_, 0.4 mM MgSO_4_.7H_2_O, 17 mM NaCl, pH 7.5, containing 0.2% glucose) and adjusted to OD_600_ 1.0 in mC medium. Saliva‐coated cover slips were prepared as above in 12‐well plates (Greiner) before bacterial cell suspension (OD_600_ 0.5) was added to each well. The plates were incubated at 37°C for 1 hr with shaking at 50 r.p.m. The cell suspensions were removed and the cover slips washed twice in mC medium. Fresh mC medium was then added to each well and the plates were incubated at 37°C for a further 15 hr. Cover slips were then removed, washed twice in PBS, and stained with crystal violet as described above.

### 4.15 Recombinant protein binding assays

ECM proteins in coating buffer (50 μl) were added to wells of a high‐binding 96‐well plate (Immulon 2HB) and incubated for 16 hr at 4°C. Wells were washed once in TBSC and non‐specific binding sites were blocked with 3% BSA in TBSC for 1 hr at 37°C. Wells were washed once in TBSC and recombinant protein (0–5 μg) diluted in TBSC was applied to the wells and incubated for 1 hr at 37°C. Unbound protein was removed and wells washed once in TBS. Primary antibody diluted in TBST was added to the wells and incubated for 1 hr at 37°C. Wells were washed twice in TBST before adding HRP‐linked secondary antibody diluted in TBST containing 3% BSA and incubating for 1 hr at 37°C. Wells were washed once in TBST, twice in TBS, and detection reagent (0.102 M Na_2_HPO_4_, 0.049 M citric acid, 0.012% H_2_O_2_, 3.7 mM *o*‐phenylenediamine) was added to wells. Plates were incubated in the dark for 10 min at room temperature, 0.56 M H_2_SO_4_ was added to stop the reactions and *A*_490_ measured. x6His‐tagged proteins were detected using anti‐tetraHis antibody (Qiagen) at 1:1000 dilution and HRP‐conjugated anti‐mouse antibody (Dako) at 1:2000 dilution. Fibrinogen was detected using rabbit anti‐human fibrinogen antibody (Dako) at 1:1000 dilution and HRP‐conjugated swine anti‐rabbit antibody (Dako) at 1:2000 dilution.

## CONFLICT OF INTEREST

The authors declare no conflicts of interest.

## Supporting information


**Figure S1.** Expression of full‐length PadA from *S. gordonii*. Cultures of *S. gordonii*UB2870 Δ*padA*/pMSP‐*padA*_*6His*_ were grown in TY‐Glc medium containing nisin (100 ng ml^−1^) at 37°C to early stationary phase. Cultures were centrifuged (12000 × g, 10 min, 4°C), the supernatants carefully removed, filtered and concentrated to 4 ml with a centrifugal filter unit. Samples were subjected to SDS‐PAGE and gels were stained with Coomassie Blue (lanes 1–3) or electroblotted onto nitrocelluloseand incubated with anti‐PadAF2 antibodies (lanes 4 and 5). Antibody binding was detected with HRP‐conjugated goat anti‐rabbit antibodies followed by ECL (Amersham) on X‐ray film. Lanes: 1, Molecular mass markers; 2, DL1; 3, UB2870; 4, DL1; 5, UB2870. PadA_*6His*_is indicated by the arrow.

Supporting info item
