# Supplementary material for: Concerted functions of Streptococcus gordonii surface proteins PadA and Hsa mediate activation of human platelets and interactions with extracellular matrix
Source: Cell Microbiol. 2016 Oct 11;19(1):e12667. doi: 10.1111/cmi.12667 (PMC5574023; doi:10.1111/cmi.12667)
Supplement: Supplementary file 1 — Supporting info item [file CMI-19-na-s001.pdf]

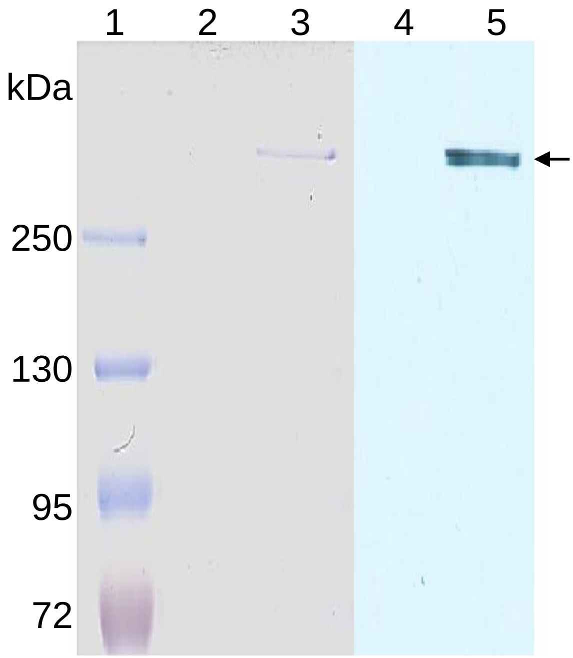

Figure S1. Expression of full-length PadA from *S. gordonii*. Cultures of *S. gordonii* UB2870 Δ*padA*/pMSP-$padA_{6His}$ were grown in TY-Glc medium containing nisin (100 ng ml^{-1}) at 37°C to early stationary phase. Cultures were centrifuged (12000 x g, 10 min, 4°C), the supernatants carefully removed, filtered and concentrated to 4 ml with a centrifugal filter unit. Samples were subjected to SDS-PAGE and gels were stained with Coomassie Blue (lanes 1-3) or electroblotted onto nitrocellulose and incubated with anti-PadAF2 antibodies (lanes 4 and 5). Antibody binding was detected with HRP-conjugated goat anti-rabbit antibodies followed by ECL (Amersham) on X-ray film.
Lanes: 1, Molecular mass markers; 2, DL1; 3, UB2870; 4, DL1; 5, UB2870. $PadA_{6His}$ is indicated by the arrow.
